# Identification of early myeloid progenitors as immunosuppressive cells

**DOI:** 10.1038/srep23115

**Published:** 2016-03-16

**Authors:** Shiming Pu, Baoxiong Qin, Huan He, Jinxi Zhan, Qiong Wu, Xinming Zhang, Liu Yang, Chunfeng Qu, Zuping Zhou

**Affiliations:** 1 School of Life Sciences, Guangxi Normal University, Guilin 541004, China; 2 Guangxi Universities Key Laboratory of Stem cell and Biopharmaceutical Technology, Guangxi Normal University, Guilin 541004, China; 3 Research Center for Biomedical Sciences, Guangxi Normal University, Guilin 541004, China; 4 State Key Laboratory of Molecular Oncology, Cancer Hospital/Institute, Chinese Academy of Medical Sciences & Peking Union Medical College, Beijing 100021, China

## Abstract

Growing evidence suggests that hematopoietic stem/progenitor cells (HSPCs), precursors of mature immune cells, may play a direct role in immunosurveillance. Early myeloid progenitors are the major components of HSPCs and they often undergo extensive expansion in stress as a result of myeloid-biased hematopoiesis. Yet, the precise function of early myeloid progenitors remains unclear. Here we show that during tumor progression, mouse granulocyte/macrophage progenitors (GMPs) but not common myeloid progenitors (CMPs) are markedly expanded within the bone marrow and blood of mice. Interestingly, both GMPs and CMPs freshly isolated from either tumor-bearing or naïve animals are capable of inhibiting polyclonal stimuli- and alloantigen-induced T cell proliferation, with tumor host-derived cells having elevated activities. Strikingly, these early myeloid progenitor cells even display much stronger suppressive capacity than the classical myeloid-derived suppressive cells. Analysis of GMPs indicates that they express iNOS and can secrete high levels of NO. Further studies unusing iNOS specific inhibitors reveal that the immunosuppression of GMPs is, to a large extent, NO-dependent. GMPs can also efficiently induce regulatory T cell development. These studies demonstrate that early myeloid progenitors can act as immunosuppressive cells. This finding provides novel insights into the functional diversity and plasticity of early myeloid progenitor cells.

Hematopoietic stem/progenitor cells (HSPCs) are a rare population of precursors responsible for continuous production of blood cells throughout life^1^,^2^. However, accumulating studies indicate that HSPCs can respond to danger signals directly^3^,^4^ and they may play an important part in the pathogenesis of various diseases, such as infection, allergy and inflammation, and cancers^5^,^6^,^7^,^8^.

A striking and common feature for HSPCs in stress as well as aging proces*s* is that they preferably undergo myeloid-biased changes^9^,^10^,^11^, which is now known to be mediated mainly by two types of surface receptors depending on stimulus inputs, cytokine receptors and toll-like receptors (TLRs) that can respectively sense systemically elevated cytokines and pathogen components^12^,^13^,^14^. Moreover, pathological conditions are often associated with a profound accumulation of myeloid cells within both the bone marrow (BM) and extramedullary tissues. This so-called “emergency” or “demand-adapted” myelopoiesis is believed to provide a protective immune response by replenishing the depleted innate myeloid cells during a pathological process^14^,^15^; yet, there are convincing evidences that the largely expanded myeloid cells may act to jeopardize host immunity, thus promoting disease development. Studies in the past twenty years have characterized well several suppressive myeloid populations, including myeloid-derived suppressive cells (MDSCs)^16^, tumor-associated macrophages^17^ and regulatory dendritic cells^18^. These cell types are now generally referred to as regulatory myeloid cells, and all of them have been related to the impaired immune function accompanying stress circumstances.

Stress-induced myeloid cell expansion is not limited merely to lineages of the later stages; rather, it happens concomitantly within the early myeloid progenitor compartment. A typical example for this is the selective expansion of granulocyte/macrophage progenitors (GMPs) occurring in most of primary human CD34^+^ acute myeloid leukemia (AML) patients^19^, which has also been recapitulated in AML-modeled mice^20^. Recently, Wu WC *et al.* further showed that the frequencies of circulating GMPs were increased four to seven fold in all types of solid tumors examined^21^, suggesting a ubiquitous event of the aberrant GMP augmentation during cancer development. In addition, the phenomenon of GMP expansion has also been documented in infection and other pathological conditions^22^,^23^,^24^. So far, however, the exact function of early myeloid progenitors or whether they, like other myeloid populations with an immunoregulatory function, act to directly modulate the immunity remains unclear.

Here, we showed that both GMPs and CMPs (common myeloid progenitors) were able to strongly inhibit polyclonal stimuli- and alloantigen-induced T cell proliferation via distinct mechanisms involving the NO signaling pathway. These studies not only demonstrated a novel role for early myeloid progenitors, but also suggest that immunosuppression might represent a shared functional property for myeloid cells at different stages of differentiation.

## Results

### Hematopoietic stem/progenitor cells undergo characteristically developmental changes during tumor progression

**Figure 1:**
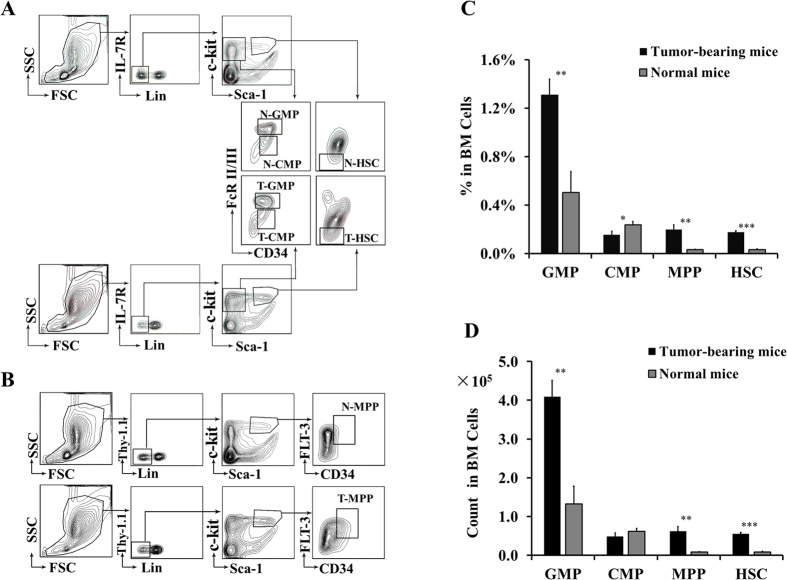
Developmental changes of hematopoietic stem/progenitor cells during tumor progression. (**A,B**) FACS analysis of BM hematopoietic stem/progenitor cell populations isolated from tumor-bearing and normal mice. Plots are examples of at least five experiments. (**C**) Frequency of indicated populations among total BM cells. (**D**) Absolute number of indicated populations in isolated BM cells. Data (mean ± SD) shown in (**C,D**) are combined from 5–8 experiments each performed with pooled samples from 4–5 mice per group. ^***^p < 0.001; ^**^p < 0.01; ^*^p < 0.05.

We first explored the developmental changes of various HSPC subsets during tumor progression. We prepared BM single cell suspensions simultaneously from tumor-bearing mice and normal mice, and analyzed them by FACS. As shown in Fig. 1, the relative percentages of T-GMP among total BM cells was increased to 1.31 ± 0.13% from 0.50 ± 0.17% of N-GMP (*P* < 0.01, Fig. 1A,C), and the absolute cell number increased to 4.09 ± 0.42 × 10^5^ from 1.32 ± 0.46 × 10^5^ of N-GMP (*P* < 0.01, Fig. 1D). A significant amplification of GMPs was also detected in blood of tumor-bearing mice (Suppl. Fig. S1). Accumulation of MDSCs is generally believed to result from a blockade of their differentiation into mature cells^16^. The marked expansion of GMPs may provide an alternative interpretation from the upstream end for how MDSCs are expanded during tumor progression. We speculate that tumor-derived factors, e.g. GM-CSF^25^, might act mainly on GMPs causing their differentiation toward MDSCs; and as such, amplification of GMPs would further aggravate the augmentation of MDSCs.

Hematopoietic stem cells (HSCs) and multipotent progenitors (MPPs) were also enormously expanded during tumor development (Fig. 1A–D). Conversely, CMPs appeared to be attenuated as reflected by their significantly reduced frequency (T-CMP vs N-CMP: 0.15 ± 0.03% vs 0.24 ± 0.03%, *P* < 0.05; Fig. 1A,C) and slightly decreased cell number (T-CMP vs N-CMP: 0.48 ± 0.10 × 10^5^ vs 0.62 ± 0.07 × 10^5^, *P* > 0.05; Fig. 1D). Tumor-trigged CMP downregulation has also been reported by other groups^20^,^21^.

### Early myeloid progenitors strongly inhibit T cell proliferation *in vitro*

**Figure 2:**
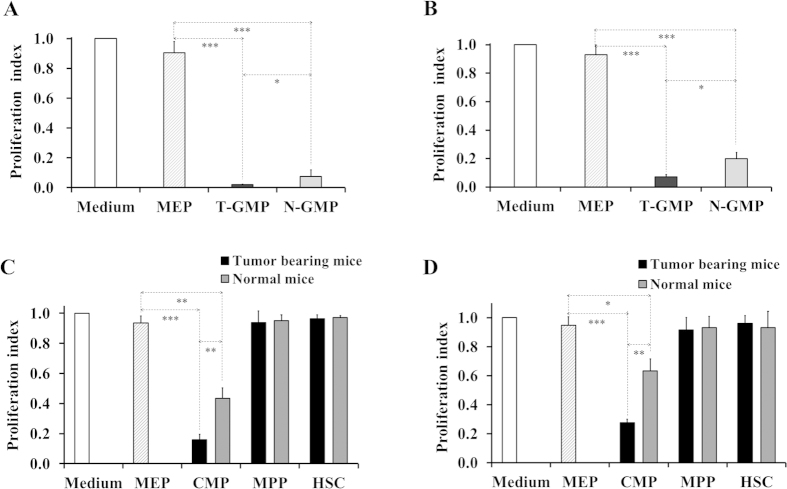
Potent suppression of T cell proliferation by early myeloid progenitor cells. (**A,C**) Inhibition of non-specific T cell proliferation by GMPs and CMPs. 2 × 10^5^ CFSE-labeled B6 splenocytes were cultured with or without indicated BM populations at a 1:1 (**A**) or 1:8 (**C**) ratio of BM cells vs splenocytes for 3 days in the presence of anti-CD3/anti-CD28 antibodies. (**B,D**) Inhibition of antigen-specific T cell proliferation by GMPs and CMPs. Cell culture procedure was similar to that as in Fig. 1A, except that mitomycin C-treated BALB/c splenocytes (2 × 10^5^) instead of anti-CD3/anti-CD28 antibodies were used as stimulators and cells incubated for 96 hrs. Data shown are mean ± SD of triplicate samples and representative of at least five experiments each performed with pooled samples from 4–5 mice per group. ^***^p < 0.001; ^**^p < 0.01; ^*^p < 0.05.

We set out to test whether GMPs would have a suppressive function. For this, BMs were isolated from tumor-bearing and normal mice, and GMPs sorted as immunophenotypically Lin^−^IL-7R^−^Sca-1^−^c-kit^+^CD34^+^FcR II/III^+^ cells^26^,^27^. We first evaluated the influence of GMPs on non-specific T cell proliferation induced by anti-CD3/anti-CD28 antibodies. As anticipated, spleen T cells proliferated vigorously when incubated in medium with or without presence of MEPs (megakaryocyte/erythrocyte progenitors, pooled from tumor-bearing and normal mice and used as control) (Fig. 2A). However, addition of either tumor mice-derived GMPs (T-GMP) or normal mice-derived GMPs (N-GMP) to the cultures almost abrogated this T cell proliferation (Fig. 2A). Similar results were obtained when purified splenocytic CD3^+^ T cells were cocultured with GMPs (Suppl. Fig. S2A), indicating that the suppression resulted simply from GMPs added rather than other types of cells present in the splenocytes. To further assess the immunosuppressive effect of GMPs and verify the results from non-specific T cell proliferation assays, mixed leukocyte reaction (MLR) experiments were performed. Again, we detected a strong inhibition of allo-antigen-stimulated T cell proliferation by both T-GMP and N-GMP (Fig. 2B). Of note, T-GMP displayed a significantly higher activity than N-GMP in both none-specific and antigen-specific T cell proliferation assays. These data clearly indicate that GMPs are highly suppressive cells and their activities can be further potentiated under tumor conditions.

Inspired by the finding with GMPs, we subsequently examined CMPs, the first myeloid-restricted population that derives from MPPs whereas gives rise to GMPs. Using the same approaches as described above, we found that even at a low ratio of CMPs vs splenocytes (1:8), CMPs (Lin^−^IL-7R^−^Sca-1^−^c-kit^+^CD34^+^FcRII/III^lo/−^ cells^26^,^27^,^28^) from either tumor-bearing mice (T-CMP) or normal mice (N-CMP) caused a dramatic reduction in T-cell proliferation stimulated by polyclonal stimuli (Fig. 2C) or allo-antigens (Fig. 2D). This suppression was also observed with sorted splenocytic CD3^+^ T cells (Suppl. Fig. S2B). In all these experiments, T-CMP exerted a greater activity than N-CMP, similar to that seen with GMPs. To determine whether CMPs represent the most upstream suppressive population of the hematopoietic pathway, we simultaneously tested MPPs and HSCs. As shown in Fig. 2C,D, addition of either cell type did not affect the non-specific and antigen-specific T-cell proliferation. Together, these results not only demonstrated a suppressive role for CMPs, but also suggest that precusor cells within the HSPC compartment might acquire suppressive ability once, or alternatively, only after they become myeloid-restricted.

### Early myeloid progenitors are more suppressive than the classical MDSCs

**Figure 3:**
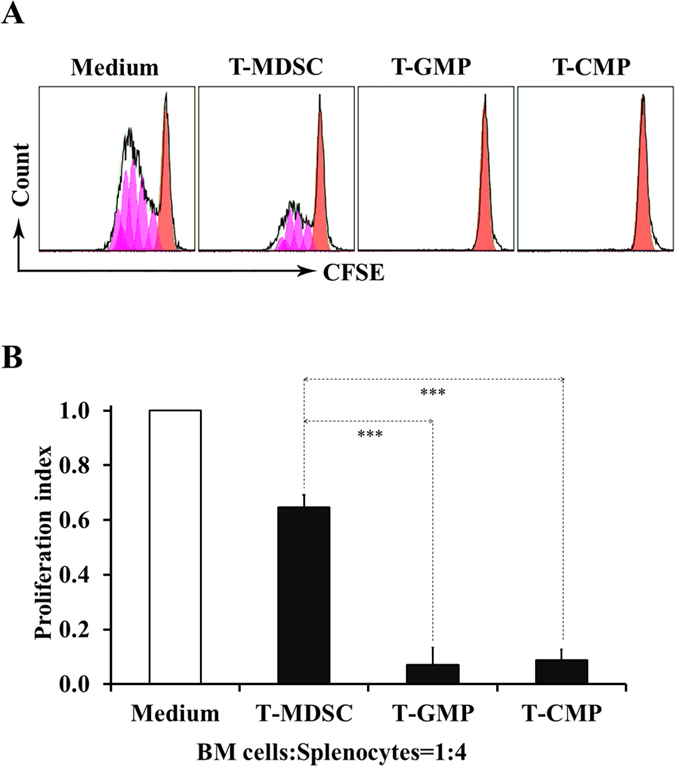
A comparison of suppressive activity between early myeloid progenitor cells and MDSCs. 2 × 10^5^ CFSE-labeled B6 splenocytes were cultured with or without indicated FACS-sorted populations from tumor-bearing mice at a 1:4 ratio of BM cells vs splenocytes for 3 days in the presence of anti-CD3/anti-CD28 antibodies, and analyzed by FACS. (**A**) Representative histograms of CFSE intensity by FACS analysis. (**B**) Proliferation index of spleen T cells. Data shown are mean ± SD of triplicate samples and representative of three independent experiments.

MDSCs are a well characterized suppressive myeloid population and they are generally defined as CD11b^+^Gr-1^+^ cells^16^. Hence, we next compared the suppressive capacities of early myeloid progenitor populations and MDSCs using tumor mice-derived cells. Strikingly, at the same cell ratio (1:4, BM cells: splenocytes), both GMPs and CMPs exhibited much stronger suppressive capacity than MDSCs (Fig. 3A). Statistical analysis indicated that the suppressive activity of MDSCs was over 8 fold lower than T-GMP (0.648 ± 0.043 vs 0.071 ± 0.063, p < 0.001) or T-CMP (0.648 ± 0.043 vs 0.087 ± 0.039, p < 0.001), whereas there was no apparent difference between the two progenitor populations (Fig. 3B). The extraordinarily inhibiting capability of early myeloid progenitors as compared to the late stage MDSCs argues strongly that their suppressive responses were generated by themselves rather than by progenies (*e.g.* MDSCs) likely derived from them.

### Early myeloid progenitors-mediated suppression depends on NO production

**Figure 4:**
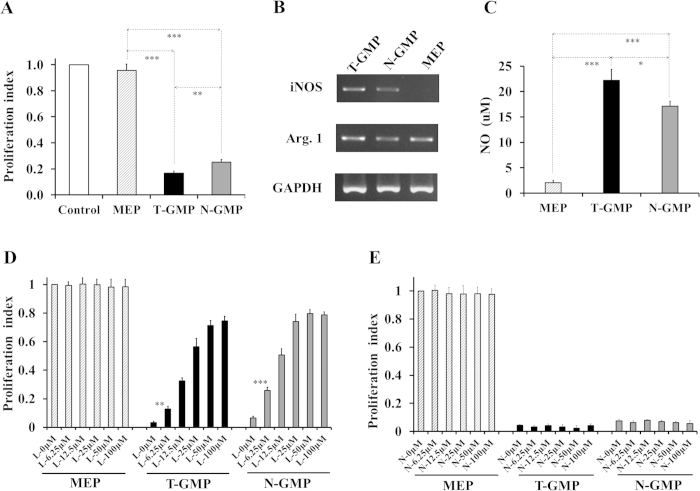
The suppressive effect of GMPs is NO-dependent. (**A**) Minimal effect of separating cell-cell contact on the GMPs-mediated suppression. 1 × 10^6^ CFSE-labeled B6 splenocytes were co-cultured with 2.5 × 10^5^ BM cells in a transwell system with splenocytes and BM cells in the lower and upper compartment, respectively. After 3-day incubation in the presence of anti-CD3/anti-CD28 antibodies, cells were recovered and analyzed by FACS. (**B**) Relative expression of iNOS and Arg.1 by GMPs. FACS-sorted cells were extracted for total RNA, and mRNA levels were determined by RT-PCR using a commercial kit. Representative data of three independent experiments are shown. (**C**) Production of NO by GMPs. Samples were collected from non-specific T cell proliferation assay cultures as described in Fig. 1A and the levels of NO in supernatants were determined using Greiss reagents per the manufacturer’s protocol. Values are mean ± SD of triplicate samples and representative of three experiments. (**D,E**) Impact of iNOS- and arginase-specific inhibitors on the suppression of GMPs. Non-specific T cell proliferation assays were performed and cells were cultured in the absence or presence of various concentration of L-NMMA (iNOS inhibitor, (**D**)) or nor-NOHA (arginase inhibitor, (**E**)). Data shown are mean ± SD of triplicate samples and representative of two reproducible experiments. ^***^p < 0.001; ^**^p < 0.01; ^*^p < 0.05.

To better understand the suppressive nature of early myeloid progenitors, we next sought to elucidate the potential underlying mechanisms. To this end, we only focused on GMPs due to the difficulty to obtain sufficient number of CMPs for assays. We first assessed whether the suppression of early myeloid progenitors is dependent upon cell-cell contact, a mechanism operative in some types of cells such as MDSCs^29^ and Tregs^30^. Using a transwell coculture system, we found that preventing the direct contact of myeloid progenitors-T cells did not alter their suppressive competency (Fig. 4A), suggesting that GMPs inhibit T cells primarily via secretion of soluble factors or molecules. NO is the key molecule mediating T-cell inhibition of distinct suppressive cells^31^,^32^,^33^. RT-PCR analysis indicated that freshly isolated GMPs expressed ample mRNA encoding iNOS (inducible nitric oxide synthase) and arginase 1 (Fig. 4B), two enzymes that share the same substrate but respectively catalyze L-arginine to generate NO or urea^34^. In consistent with their abundant expression of iNOS, both T-GMP and N-GMP secreted large amounts of NO compared to MEP controls (Fig. 4C). To demonstrate a direct involvement of NO in the suppressive effect of GMPs, T-cell proliferation was examined in the presence of specific inhibitor (*N*^*G*^-monomethyl-L-arginine, L-NMMA) for iNOS. As shown in Fig. 4D, addition of L-NMMA could effectively reverse the T-cell suppression of GMPs in a dose-dependent manner. The saturated response of L-NMMA was achieved at a concentration of 25 μM for T-GMP or 50 μM for N-GMP, which also mirrored their NO production abilities, pointing to a differed activation status between the two cell types. In contrast, inclusion of arginase inhibitor (**nor**-NOHA) in the cultures failed to change the suppressive effect of T-GMP or N-GMP (Fig. 4E), further demonstrating a critical role of NO synthesis in the process. Noteworthily, blockade of NO production could not completely overcome the suppressive effect of T-GMP or N-GMP, implying that additional pathways may participate in the inhibitive process of these cells.

### GMPs induce regulatory T cell development

**Figure 5:**
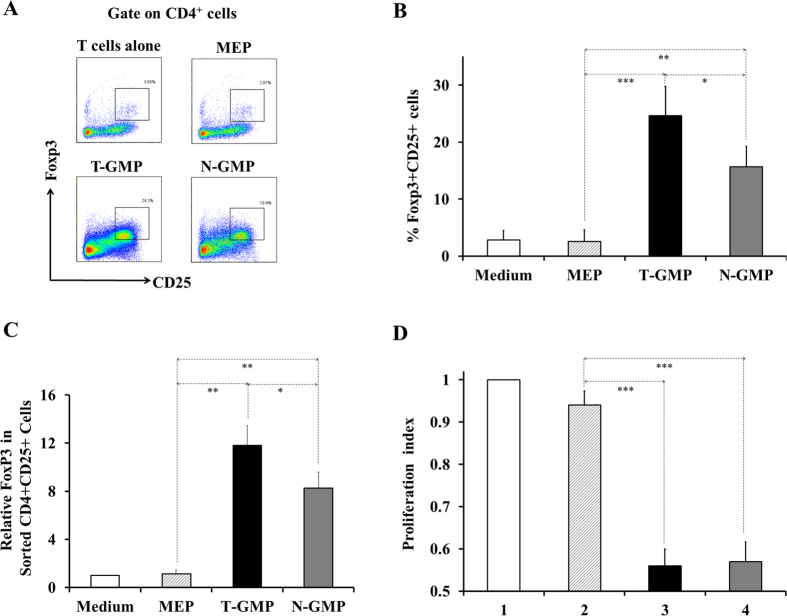
Efficient induction of Tregs by GMPs. Non-specific T cell proliferation assays were performed as described in Fig. 1A and Tregs were analyzed by FACS. In (**C,D**), the cultured cells were recovered and sorted for CD4^+^CD25^+^ cells. For analyzing Foxp3 expression (**C**), sorted CD4^+^CD25^+^ cells were used for total RNA extraction and Foxp3 expression levels determined by RT-qPCR. For assessing the functional activities of induced Tregs, sorted CD4^+^CD25^+^ cells were re-cultured with fresch CFSE-labeled splenocytes at 1:4 ratio as described above. (**A**) Example FACS dot plots gated on CD4^+^ cells. (**B**) Percentages of Foxp3^+^CD25^+^ cells induced in the CD4 T cells. (**C**) Relative Foxp3 expression in CD4^+^CD25^+^ cells recovered from indicated cultures. Foxp3 expression level in BM cells-added groups was calculated as fold of that of medium-only samples. (**D**) Inhibition of T cell proliferation by GMP-induced Tregs. 1: splenocytes only; 2, 3, 4: splenocytes + CD4^+^CD25^+^ cells recovered from original cultures with presence of MEP (2), T-GMP (3), or N-GMP (4). Data shown are mean ± SD of triplicate samples from three (**A,B**) or two (**C,D**) independent experiments^***^p < 0.001; ^**^p < 0.01; ^*^p < 0.05.

Different immunosuppressive myeloid populations including MDSCs have been shown to be capable of inducing Tregs and employ them as an indirect mechanism to assume their suppression^35^,^36^,^37^. To test whether this pathway is potentially involved in the suppressive effect of early myeloid progenitors, we assessed the ability of GMPs to induce Treg development. Remarkably, when cocultured with splenocytes *in vitro*, GMPs stimulated a significant proportion of CD4 T cells to become Foxp3^+^CD25^+^ cells (Fig. 5A), and a higher level of induction was detected with cells from tumor-bearing mice (T-GMP) than those from normal mice (Fig. 5B). The varied expression of Foxp3 in different sets of cultures was confirmed by FACS-sorting CD4^+^CD25^+^ cells and analyzing them for Foxp3 level by RT-qPCR (Fig. 5C). More interestingly, when re-incubated with fresh splenocytes, CD4^+^CD25^+^ T cells purified from GMP cultures showed a much stronger suppression than those from MEP cultures (Fig. 5D), matched well to their abundances of Foxp3^+^cells among the respective CD4^+^CD25^+^ T cell populations, indicating that GMP-induced Foxp3^+^CD25^+^ cells were functionally competent Tregs. These results suggest that early myeloid progenitors might also act to suppress T-cells indirectly through induction of Tregs.

## Discussion

We have provided firm evidence that early myeloid progenitors can act as immunosuppressive cells in addition to being hematopietic precursors. With well-established experimental systems, we showed that mouse GMPs and CMPs, two earliest populations of the myelopoietic tree, exhibited robust suppression against both non-specific and antigen-specific T-cell proliferation. Furthermore, we demonstrated, by using GMPs as an example, that the immunosuppression of early myeloid progenitors was NO-dependent but could not be fully accounted for by this single pathway, since at the saturated concentration of iNOS inhibitors they still retained around 30% of suppressive capacity. Early myeloid progenitors also could efficiently induce Treg development and expressed mRNA encoding Th2 cytokines IL-4, IL-10 and IL-13 (Suppl. Fig. S3). It remains to be determined whether these molecules or pathways are virtually or to what extent involved in the suppressive process of early myeloid progenitors.

Somewhat unexpectedly, in this study we consistently observed that both GMPs and CMPs recovered from naive mice also possessed suppressive ability albeit with a lower activity compared to their tumor-activated counterparts. Notably, coinciding with their suppressive capabilities, normal mice-derived GMPs produced less NO than those from tumor mice, which was confirmed by their differed requirement of L-NMMA dose to overcome the suppressive effect as demonstrated in assays with iNOS inhibitors. Similar differences were also detected in induction of Tregs. It is likely that early myeloid progenitors might present in steady status with a physiological level of suppressive activity, which could be further exacerbated under stress. It would be interesting and necessary to determine whether the distinct pathways involved are subject to regulation by the same or different mechanism in future study. The inherently suppressive progenitor cells, as compared to other immunoregulatory cell types within the BM, such as Tregs, MDSCs, and MSCs (mesenchymal stem cells)^38^, may formulate an additionally but also physically advantageous layer of protection against immune reactions to ensue the orderly commitment and generation of hematopoietic cells.

An immunosuppressive function has been recognized for various myeloid populations. Among the most scrutinized are MDSCs, a heterogeneous population consisting of primarily monocytic- and granulocytic-like cells^16^. MDSCs are defined on the basis of two hallmark features: immature and myeloid origin^39^, and therefore it is widely considered that MDSCs may encompass myeloid populations at earlier stages^29^,^39^,^40^. Interestingly, a recent study demonstrated that promyelocytes, the precursors of granunocytes, were also immunosuppressive^41^. Although the relationship of early myeloid progenitors and MDSCs still needs further evaluation given their remarkable difference in suppressive capacity, the data presented here and those mentioned above undoubtedly provide further evidence that immunosuppression is likely a shared functional property for immature myeloid cells. In addition, that early myeloid progenitors (at least for GMPs) can function as suppressive cells is also supported by several other lines of studies. First, we previously demonstrated that a GMP-like cell population derived from either mouse ES cells or BM HSCs can prevent effectively the development of graft-versus-host disease (GvHD) following adoptive transfer^33^. Furthermore, like the *ex vivo* GMPs tested herein, those *in vitro*-generated cells also display much stronger suppressive capacity than CD11b^+^Gr-1^+^ MDSCs in T-cell proliferation assays^33^. Second, Young MR *et al.* reported that tumor-induced suppressive cells within BM phenotypically resemble the GMP cells in a murine LLC model^42^. Third, during cancer development GMPs are selectively enriched in tumor sites, and their levels correlate positively with disease progression^21^, suggesting a negative control of GMPs in tumor immunity.

HSPCs are increasingly appreciated as both precursors and effectors. An interesting and important aspect emerging from this study is that our data may provide valuable clues to dissecting and understanding the potentially novel functions of HSPCs. For instance, an immunosuppressive nature of HSPCs has long been implicated by their potent induction of transplantation tolerance^43^,^44^. Our finding of CMPs and GMPs (but not HSCs and MPPs) being suppressive cells offers a plausible explanation concerning the cellular basis behind the immunosuppression of HSPCs, and suggests that their suppressive effect might be contributed exclusively by the myeloid progenitor subpopulations. Besides, numerous studies have demonstrated that circulating HSPCs harbor a population called innate Th2 cytokine-producing cells critical for inflammatory diseases^45^. It will be interesting to determine in future if there are any functional or even lineage links between these proinflammatory cells and the suppressive myeloid progenitors.

In summary, we have demonstrated for the first time that early myeloid progenitors can act as immunoregulatory cells. This finding provides unique insights into the functional diversity and plasticity of early myeloid progenitor cells; and particular significantly, it also raises the possibility that harnessing the earliest populations of myelopoietic pathway may prove useful in generating novel therapeutic interventions for myeloid cell-associated immune dysfunction in pathological settings.

## Methods

### Animals and reagents

Female C57BL/6 (B6) and BALB/c mice at 8–10 weeks of age were purchased from SLAC Ltd. Animals were maintained in the Guangxi Normal University Laboratory Animal Center and handled in accordance with the institution’s guidelines. All experimental protocols were approved by the Guangxi Animal Management Committee of Guangxi S&T Department (Project Number syxk (Gui) 2013-0001). Fluorescence-conjugated anti-mouse mAbs anti-c-kit(ACK2), anti-Sca-1(D7), anti-CD34(RAM34), anti-IL-7R(A7R34), anti-FcR II/III(93), anti-Flt3(A2F10), anti-Thy-1.1(HIS51), and their isotype ctrl antibodies (except those for anti-IL-7R and anti-Thy-1.1) were obtained from affymetrix eBioscience. Anti-mouse Lineage cocktail (containing anti-CD3, Ly-6G/Ly-6C, anti-CD11b, anti-CD45R/B220 and anti-TER-119) with isotype ctrl was from Biolegend. Mitomycin C was obtained from Solarbio.

### Tumor model

Lewis Lung Carcinoma (LLC) cell line was obtained from the State Key Laboratory of Molecular Oncology, and tumor model was established as previously described^33^. Briefly, C57BL/6 mice were injected s.c. with 5 × 10^5^ tumor cells and control mice injected with PBS. Mice with a tumor size of ~1.5 cm were used.

### Isolation and sorting of BM cells

Mice were sacrificed 4 weeks post inoculation, and tibia and fibula harvested. BMs were prepared as single cell suspension after depletion of red blood cells. Cell populations were sorted using FACSaria II according to previous reports^26^,^27^,^28^. The gating strategies were shown as in Fig. 1 and Suppl. Fig. S4, and cells with a purity of over 95% were used (Suppl. Fig. S4).

### T-cell suppression/proliferation assay

T cell proliferation assays were carried out as described previously^33^. Cell proliferation was assessed using FACSVerse and data analyzed with FlowJo. Proliferation index was applied to reflect T-cell proliferation, and the value (percentage of CFSE-diluted T cells among total CFSE-labeled splenocytes or T cells) was designed as 1 for cultures with medium only (DMEM + 10%FBS + 1%Pen/Strep), and those for other culture conditions were normalized to the medium only group.

### NO measurement

Supernatants from none-specific T cell proliferation assay cultures were collected and stored at −86 °C. NO was measured by Greiss reagents (Sigma-Aldrich) per the manufacturer’s protocol.

### Regulatory T cells induction

Cell culture procedure was the same as for non-specific T cell proliferation assay. Tregs were analyzed by FACS using a mouse Treg staining kit (affymetrix eBioscience).

### RT-PCR and RT-qPCR

Total RNA was extracted using TRIzol reagent (Invitrogen). A one-step RT-PCR kit (Qiagen) was utilized for reverse transcription of RNA and amplification of cDNA. For quantative RT-PCR (**RT-qPCR),** total RNA was prepared using RNAprep Pure Micro Kit (TIAN GEN) and reversely transcriped into cDNA with the FastQuant RT Kit (TIAN GEN). Real-time PCR was performed on the ABI 7500 Fast instrument. GAPDH was used as the reference genes for normalization of FoxP3 expression. Gene primer pairs used were attached in Suppl. Table S1.

### Statistical analysis

All data were analyzed with SPSS 19.0 using two-tailed unpaired Student *t*-test.

## Additional Information

**How to cite this article**: Pu, S. *et al.* Identification of early myeloid progenitors as immunosuppressive cells. *Sci. Rep.* **6**, 23115; doi: 10.1038/srep23115 (2016).

## Supplementary Material

Supplementary Information
