# Supplementary material for: Identification of early myeloid progenitors as immunosuppressive cells
Source: Sci Rep. 2016 Mar 16;6:23115. doi: 10.1038/srep23115 (PMC4793235; doi:10.1038/srep23115)
Supplement: Supplementary Information [file srep23115-s1.pdf]

Supplementary Information

Identification of early myeloid progenitors as immunosuppressive cells

Shiming Pu[1,2], Baoxiong Qin[1], Huan He[4], Jinxi Zhan[1], Qiong Wu[1,2,3], Xinming Zhang[1,3], Liu Yang[1,2,3], Chunfeng Qu[4], Zuping Zhou[1,2,3*]

[1]School of Life Sciences, Guangxi Normal University, Guilin 541004, China; [2]Guangxi Universities Key Laboratory of Stem cell and Biopharmaceutical Technology, Guangxi Normal University, Guilin 541004, China; [3]Research Center for Biomedical Sciences, Guangxi Normal University, Guilin 541004, China; [4]State Key Laboratory of Molecular Oncology, Cancer Hospital/Institute, Chinese Academy of Medical Sciences & Peking Union Medical College, Beijing 100021, China

[*]Corresponding author

Supplementary Table S1. Gene primers used in the study

Gene	Accession number	Primer Sequence	PCR Product size (bp)
iNOS	NM_010927.3	F:TGTTCTCAGCCCAACAATACA<br>R: CCCAGTTTTTGATCCTCACATA	152
Arg-1	NM_007482.3	F: CTCCAAGCCAAAGTCCTTAGAG<br>R: AAGGAGCTGTCATTAGGGACAT	186
IL-4	NM_021283.2	F: ATCATCGGCATTTTGAACGAGG<br>R:TGCAGCTCCATGAGAACACTA	200
IL-10	NM_010548.2	F: AGCCTTATCGGAAATGATCCAGT<br>R: GGCCTTGTAGACACCTTGGT	229
IL-13	NM_008355.3	F:ATATATGGAAGAATGGCCTGTTA<br>R:TTATTTCCGGTTTCTAGTTTGAC	116
TGF-β	NM_011577.1	F:GTGGTATACTGAGACACCTTGG<br>R:CCTTAGTTTGGACAGGATCTGG	374
FoxP3	NC_000074.5	F: CCCATCCCCAGGAGTCTTG<br>R: CCCATCCCCAGGAGTCTTG	339
GAPDH	NM_008084.2	F:GCTGCCCAGAACATCATCCCT<br>R: TGAAGTCGCAGGAGACAAC	256

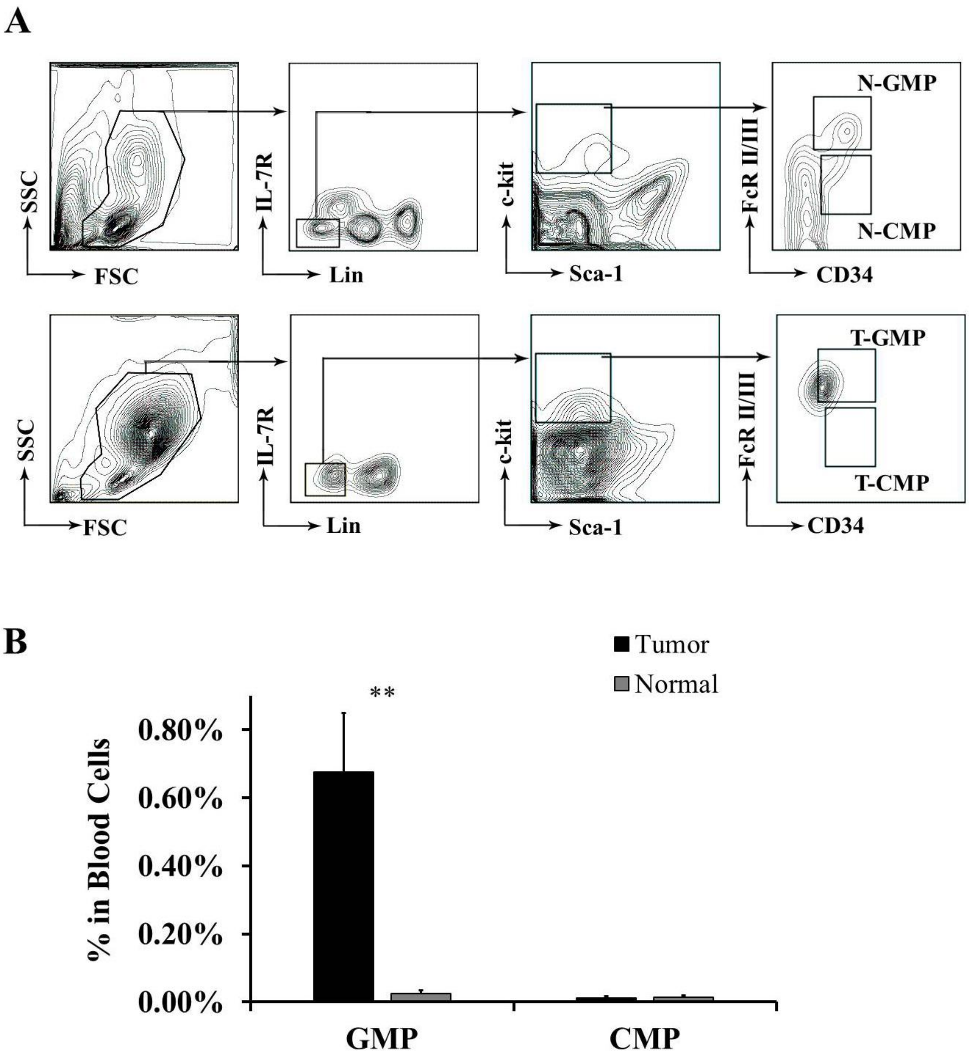

Supplementary Figure S1. Marked expansion of GMPs in blood of tumor-bearing mice. GMPs are markedly expanded in blood of tumor-bearing mice ($p<0.01$), and no significant difference of CMP frequencies was seen between normal and tumor-bearing mice ($p>0.05$). Blood samples were collected from the eyes of normal and tumor-bearing mice. After depletion of red blood cells, samples were stained with indicated Abs and analyzed by FACS. Example plots were shown in **(A)**, and the cell frequencies (mean ± SD) shown in **(B)** were combined data from two experiments each performed with three mice per group. **$p<0.01$**.**

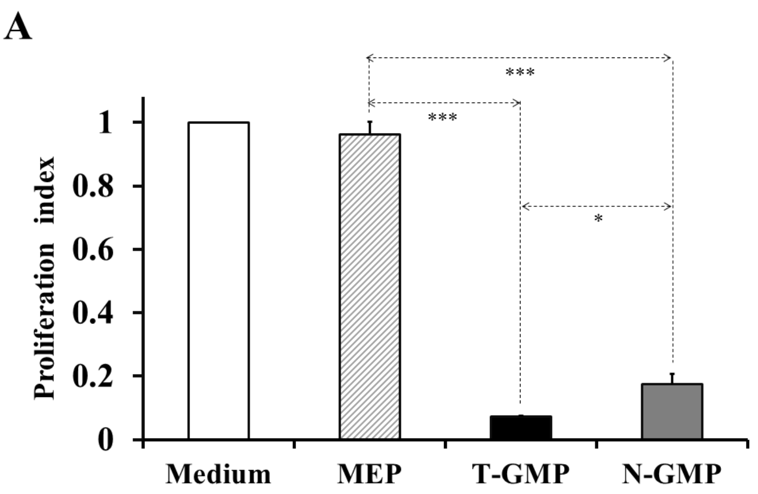

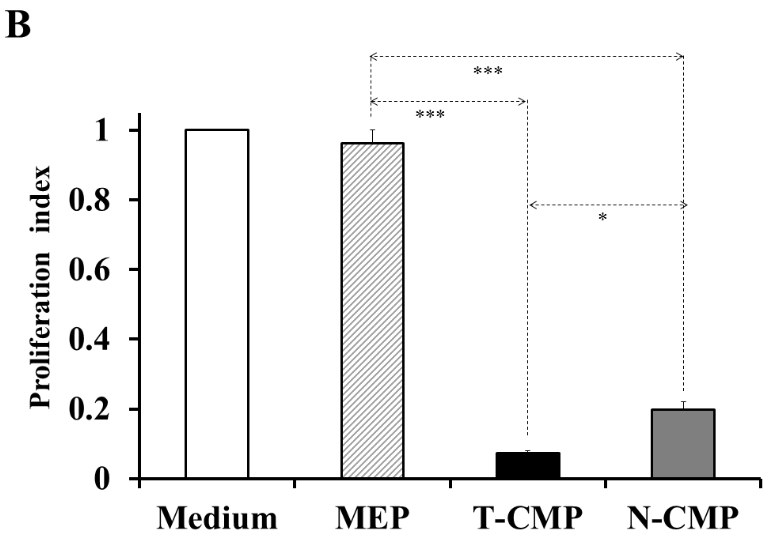

Supplementary Figure S2. **Inhibition of splenocytic T cell proliferation by GMPs (A) and CMPs (B)**. B6 mouse splenocytic $CD3^+$ T cells were negatively selected and purified using the Mouse T Cell Enrichment Columns (CA#: MTCC-5/10/25, R&D Systems). After CFSE labeling, 2×10^4 T cells were cocultured with or without indicated BM populations at a 1:2 ratio of BM cells vs T-cells for 3 days in the presence of anti-CD3/anti-CD28 antibodies. Data shown are mean ± SD of triplicate samples and representative of3(A) or 2 (B) reproducible experiments. ***, $p<0.001$; *, $p<0.05$.

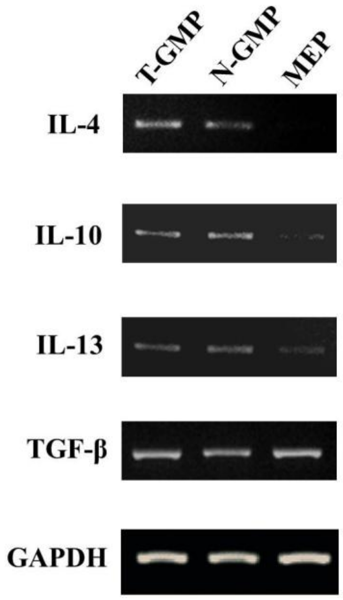

Supplementary Figure S3. Relative expression of mRNA encoding cytokines IL-4, IL-10, IL-13, and TGF-β by GMPs. FACS-sorted cells were extracted for total RNA, and mRNA levels were determined by RT-PCR using a commercial kit. Representative data of two independent experiments are shown. T-, tumor mice; N, normal mice.

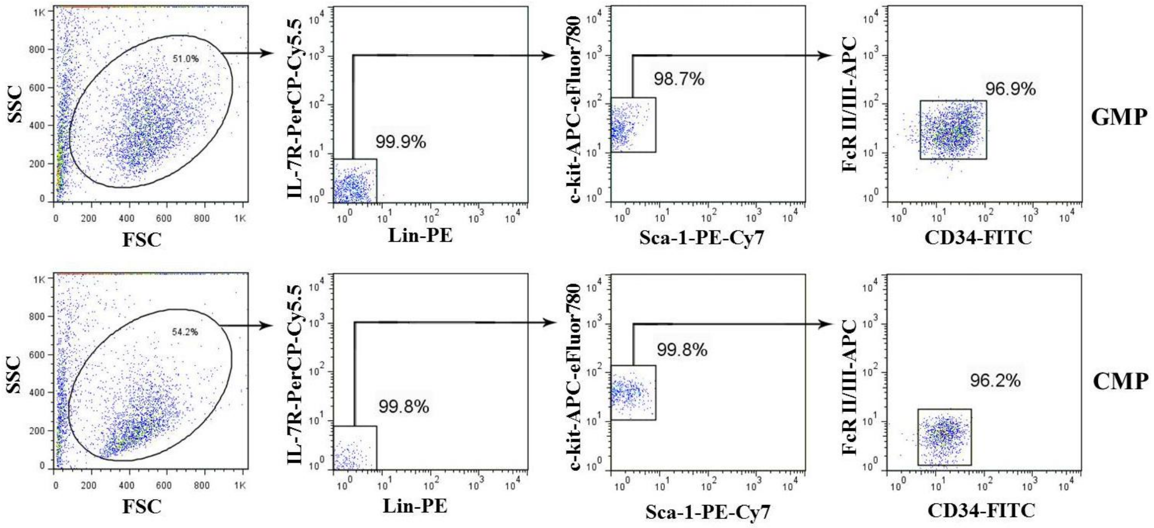

Supplementary Figure S4. An examination of purities for FACS-sorted cells. Sorted cells were directly subjected to analysis using FACSVerse. Data shown are representative of at least three experiments.
